# Nebivolol suppresses glioblastoma progression via dual modulation of mitochondrial metabolism and AKT/mTOR/4EBP1 signaling axis

**DOI:** 10.1371/journal.pgen.1012120

**Published:** 2026-04-24

**Authors:** Lingni Zhou, Hongyu Che, Hongyan Jiang, Luxin Yin, Yongang Jiang, Yuhui Zhang, Haoran Liang, Rutong Yu, Xu Zhang, Xuejiao Liu

**Affiliations:** 1 Department of Neurosurgery, Affiliated Hospital of Xuzhou Medical University, Xuzhou, Jiangsu, China; 2 Insititute of Nervous System Diseases, Xuzhou Medical University, Xuzhou, Jiangsu, China; 3 Department of Critical Care Medicine, Yancheng Third People’s Hospital, Yancheng, Jiangsu, China; Tulane University School of Medicine, UNITED STATES OF AMERICA

## Abstract

Emerging evidence reveals the pivotal involvement of mitochondrial metabolic dysregulation in glioblastoma (GBM) pathogenesis, considering mitochondrial metabolism as a potential therapeutic target. Nebivolol, a third-generation β-adrenergic receptor antagonist clinically employed in cardiovascular diseases, has recently exhibited notable anti-neoplastic properties. Nevertheless, its therapeutic efficacy and mechanistic underpinnings in GBM remain largely unexplored. In this investigation, we comprehensively assessed the impact of nebivolol on GBM cellular proliferation and elucidated its molecular mechanisms. Our findings revealed that nebivolol markedly suppressed the proliferation and clonogenic abilities of multiple GBM cell lines, concomitant with cell cycle arrest and apoptotic induction. Mechanistically, nebivolol impaired mitochondrial respiratory chain complex I activity, diminished adenosine triphosphate (ATP) synthesis, and augmented ROS production, collectively precipitating neoplastic cell apoptosis. Furthermore, nebivolol attenuated AKT/mTOR/4EBP1 signaling cascade activation, thereby impeding GBM malignant proliferation. *In vivo* studies corroborated these observations, demonstrating that nebivolol administration significantly attenuated orthotopic GBM xenograft progression and extended survival in tumor-bearing murine models. This study delineates a novel dual mechanism whereby nebivolol exerts anti-GBM effects through concurrent modulation of mitochondrial bioenergetics and AKT/mTOR/4EBP1 signaling transduction. These results provide robust preclinical evidence supporting nebivolol’s clinical repurposing for GBM therapy.

## Author summary

Glioblastoma (GBM) is the most malignant and aggressive form of brain tumor, characterized by limited therapeutic options and poor prognosis. Despite continuous advances in ongoing research, effective therapeutic strategies remain scarce. In this study, we investigated the repurposing potential of nebivolol, a commonly used antihypertensive agent, for GBM therapy. Our findings demonstrate that nebivolol markedly suppresses GBM tumor growth, concomitant with cell cycle arrest and apoptotic induction. Mechanistically, we elucidated a dual mechanism of action: nebivolol disrupts mitochondrial function to deprive tumor cells of essential energy supplies, while simultaneously inhibiting the oncogenic AKT/mTOR/4EBP1 signaling pathway. This combined effect efficiently induces tumor cell death and substantially impedes disease progression. In an orthotopic xenograft mouse model, we demonstrated that nebivolol treatment effectively suppressed intracranial tumor growth and extended survival in tumor-bearing mice. Given its established clinical safety profile and high permeability of the blood-brain barrier, nebivolol represents a promising candidate for drug repurposing in GBM, offering a potential pathway toward an urgently needed therapeutic option for patients with this devastating malignancy.

## Introduction

Glioblastoma multiforme (GBM) is the most common and aggressive malignant brain tumor in adults, characterized by a high mortality rate and frequent recurrence. GBM accounts for 57% of all gliomas [1,2]. The current standard treatment protocol involves multimodal therapy, including surgical resection combined with radiotherapy and temozolomide chemotherapy. Despite these interventions, patient prognosis remains poor, with a 5-year survival rate of only 6.81% [3] and a median overall survival of 14.6 to 20.5 months [2]. Although some progress has been made in GBM research, therapeutic efficacy remains limited, underscoring the urgent need to explore novel treatment strategies [3,4].

Cellular energy metabolism is primarily divided into two pathways: glycolysis and mitochondrial metabolism [5–7]. Mitochondria, as the core of cellular energy supply [8], regulate oxidative phosphorylation (OXPHOS), apoptosis, cell signaling, and the generation and regulation of reactive oxygen species (ROS) [9–12]. OXPHOS is a central component of cellular metabolism [8,13], transferring electrons provided by NADH and FADH2 in the tricarboxylic acid (TCA) cycle to adenosine diphosphate (ADP) through the mitochondrial electron transport chain (ETC) to generate adenosine triphosphate (ATP) [9]. The ETC comprises four protein complexes: NADH dehydrogenase (complex I), succinate dehydrogenase (complex II), cytochrome c reductase (complex III), and cytochrome c oxidase (complex IV) [14]. Among these, complex I is the largest membrane protein complex in the respiratory chain [15]. Inhibition of its function disrupts the mitochondrial oxidative respiratory chain, reduces ATP production, and suppresses tumor initiation and progression [16]. Additionally, complex I is a major source of ROS. While moderate ROS levels can activate signaling pathways and promote cancer cell proliferation, excessive ROS irreversibly damages cellular macromolecules, including proteins, lipids, and nucleic acids, ultimately leading to cell death [17,18]. Metformin, a drug widely used to treat diabetes, exerts anti-tumor effects by inhibiting ubiquinone reduction, reducing complex I activity, and inducing ROS production [19]. Thus, targeting mitochondrial metabolism represents a promising therapeutic strategy for tumors.

The β1-adrenergic receptor (β1-AR) is an important membrane receptor belonging to the G protein coupled receptors, encoded by *ADRB1* [20]. β1-AR contributes to tumor cell proliferation, invasion, angiogenesis, and microenvironment remodeling in various cancers, including glioma, by triggering the classical cyclic adenosine monophosphate (cAMP)/protein kinase A (PKA) and mitogen‑activated protein kinase (MAPK) pathways [21,22]. Nebivolol, a third-generation β1-AR blocker with vasodilatory effects, is approved by the U.S. Food and Drug Administration for the treatment of cardiovascular and cerebrovascular diseases [23,24]. Its safety has been well-established in clinical practice. As a lipophilic small molecule, nebivolol can penetrate the blood-brain barrier and exert pharmacological effects in the central nervous system [25]. Extensive clinical data indicate that nebivolol is well-tolerated, has minimal side effects, and exhibits potential anti-tumor activity. In various cancer models, including oral squamous cell carcinoma, colon cancer, and breast cancer, nebivolol inhibits tumor progression by suppressing mitochondrial activity [7,26]. For instance, in oral squamous cell carcinoma, nebivolol inhibits tumor growth by blocking the PERK/eIF2α signaling pathway and inducing endoplasmic reticulum (ER) stress. These findings suggest that nebivolol’s anti-tumor effects, mediated through β1-AR blockade, may offer new therapeutic avenues for GBM. However, its anti-tumor activity and mechanisms of action in GBM remain poorly understood.

This study evaluated the effects of nebivolol on GBM cell growth and xenograft tumor models, further exploring the molecular mechanisms underlying its anti-tumor effects, including the inhibition of proliferation and induction of apoptosis.

## Results

### Nebivolol, a β1-adrenergic receptor blocker, significantly inhibits GBM cell proliferation and clonogenicity

**Fig 1:**
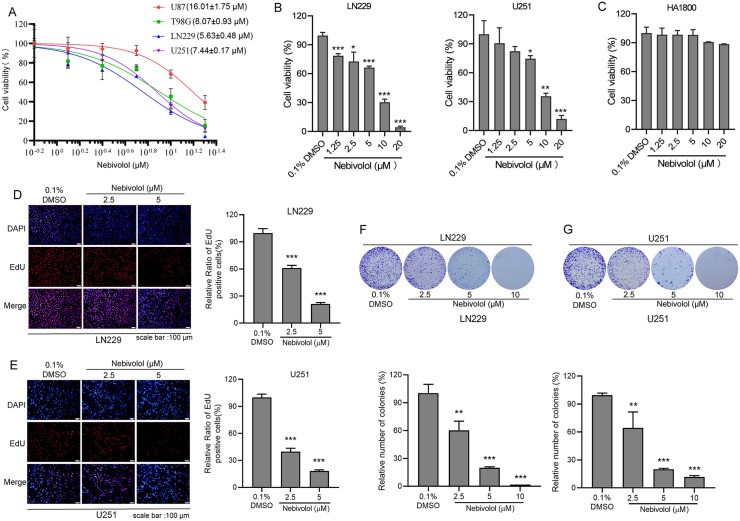
Nebivolol significantly inhibits the proliferation and clonogenic potential of GBM cells. **(A)** Cell viability analysis demonstrating the dose-dependent effect of nebivolol (0.1% DMSO as vehicle control) on four GBM cell lines following 72-hour treatment. **(B-C)** Quantitative assessment of cell viability using CCK-8 assay in LN229, U251 and HA1800 cells after nebivolol treatment. **(D-E)** Representative images and quantitative analysis of EdU incorporation assay in LN229 and U251 cells treated with indicated concentrations of nebivolol for 24 hours, scale bar: 100 μm. **(F-G)** Dose-dependent inhibition of colony formation capacity in LN229 and U251 cells following nebivolol treatment, with quantitative analysis of colony formation assay. Data are expressed as means ± SD. Statistical significance was determined by comparison with the 0.1% DMSO control group (*p < 0.05, **p < 0.01, ***p < 0.001).

To investigate the anti-tumor effects of nebivolol, a β1-adrenergic receptor blocker [24], we evaluated its growth inhibitory activity on GBM cell lines. Nebivolol exhibited significant concentration-dependent inhibitory effects on all four cell lines, though sensitivity varied among them (IC_50_: from 5.63 ± 0.48 to 16.01 ± 1.75 μΜ) (Fig 1A and 1B). The statistical results showed that U251, LN229, and T98G cells had comparable IC_50_ values (*P* > 0.05), whereas U87 cells exhibit significantly higher IC_50_ values compared to each of the other three lines (*P* < 0.001) (date not shown). In addition, Nebivolol exerted no significant inhibition on HA1800, a normal human astrocyte cell line (Fig 1C). LN229 and U251 cells were selected for further experiments.

To assess the impact of nebivolol on GBM cell proliferation, U251 and LN229 cells were treated with nebivolol for 24 hours, followed by 5-ethynyl-2′-deoxyuridine (EdU) incorporation assays. The results revealed that treatment with 2.5 µM nebivolol reduced the average proliferation rates of LN229 and U251 cells to 61% and 40%, respectively, compared to the control group. At a higher concentration of 5 µM, the proliferation rates were further suppressed to 21% and 18%, respectively, indicating a dose-dependent inhibition of GBM cell proliferation (Fig 1D and 1E).

To evaluate the long-term inhibitory effects of nebivolol on GBM cell growth, colony formation assays were performed. Nebivolol treatment significantly impaired the clonogenic capacity of GBM cells in a dose-dependent manner (Fig 1F and 1G). Collectively, these results demonstrate that nebivolol effectively suppresses GBM cell proliferation and clonogenicity, highlighting its potential as a therapeutic agent for GBM.

### Nebivolol enhances Caspase-3/7 activity and induces apoptosis in GBM cells

**Fig 2:**
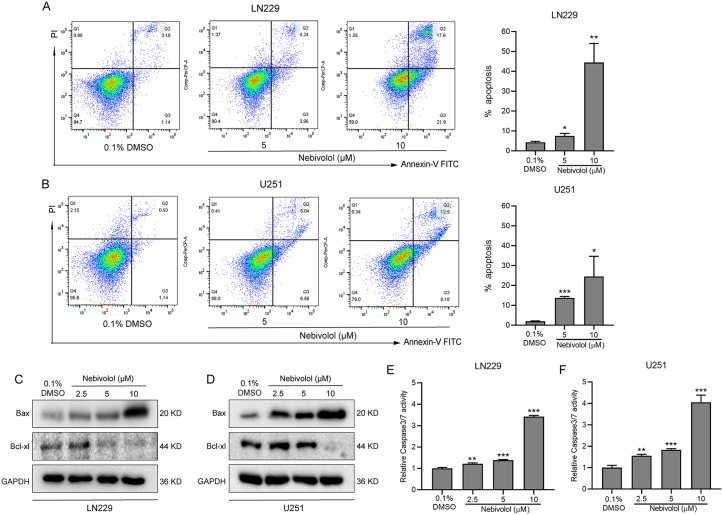
Nebivolol activates caspase 3/7 and induces apoptotic signaling in GBM cells. **(A-B)** Flow cytometric analysis of apoptosis in LN229 and U251 cells treated with 0.1% DMSO (vehicle control) or nebivolol for 24 hours, followed by Annexin V/PI double staining. The bar graph represents the total apoptotic cell population, including early apoptotic (Annexin V-positive only) and late apoptotic (Annexin V/PI double-positive) cells. **(C-D)** Western blot analysis of apoptosis-related proteins (Bax and Bcl-xL) in LN229 and U251 cells treated with increasing concentrations of nebivolol. β-actin was used as a loading control. **(E-F)** Caspase 3/7 activity was measured using the Caspase-Glo 3/7 assay in LN229 and U251 cells after 24 hours treatment with 0.1% DMSO or nebivolol. Data are presented as mean ± SD. Statistical significance was determined by comparison with the vehicle control group (*p < 0.05, **p < 0.01, ***p < 0.001).

To investigate the pro-apoptotic effects of nebivolol on GBM cells, LN229 and U251 cells were treated with varying concentrations of nebivolol (5 and 10 µM) for 24 hours, followed by apoptosis analysis using flow cytometry. Compared to the control group, nebivolol treatment significantly increased the apoptosis rate in a dose-dependent manner. At 5 µM, the average apoptosis rates in LN229 and U251 cells increased to 7.49% and 13.7%, respectively; at 10 µM, the rates further increased to 44.4% and 24.54%, respectively (Fig 2A and 2B), indicating that nebivolol effectively induces apoptosis in GBM cells.

To explore the underlying mechanisms, we examined the expression levels of the anti-apoptotic protein Bcl-xl and the pro-apoptotic protein Bax in LN229 and U251 cells using Western blotting. With increasing concentrations of nebivolol, Bcl-xl expression was progressively downregulated, while Bax expression was upregulated (Fig 2C and 2D). Furthermore, caspase-3/7 activity was assessed. Nebivolol treatment resulted in a dose-dependent increase in caspase-3/7 activity in both LN229 and U251 cells (Fig 2E and 2F). Collectively, these findings demonstrate that nebivolol promotes apoptosis in GBM cells by upregulating Bax, downregulating Bcl-xl, and enhancing caspase-3/7 activity, highlighting its potential as a therapeutic agent for GBM.

### Nebivolol induces cell cycle arrest at G0/G1 phase in GBM cells

**Fig 3:**
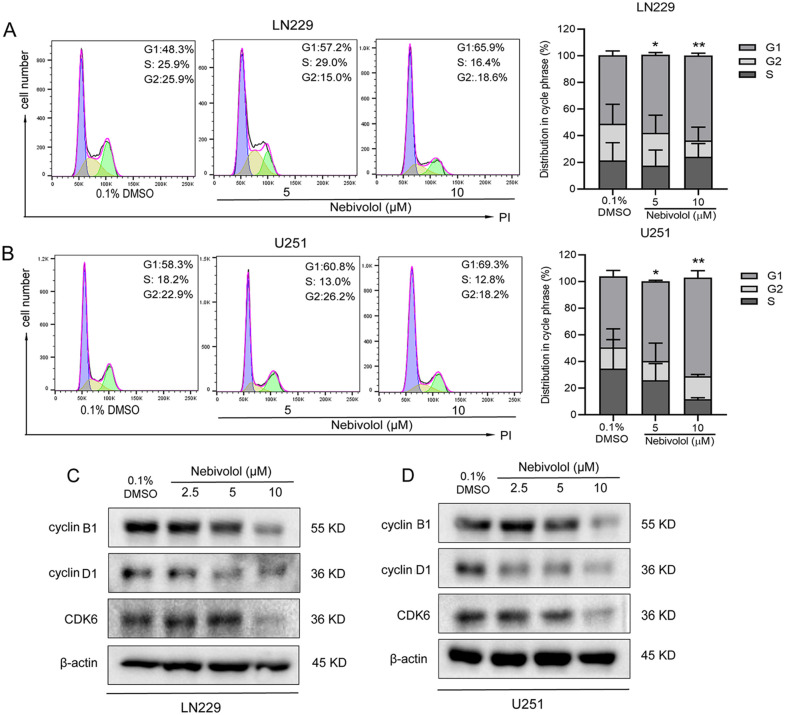
Nebivolol induces G0/G1 phase cell cycle arrest in GBM cells. **(A-B)** Cell cycle distribution analysis by flow cytometry in LN229 and U251 cells treated with nebivololor 0.1% DMSO (vehicle control) for 24 hours. Representative histograms depict the cell cycle profiles, and quantitative analysis shows the percentage of cells in G0/G1, S, and G2/M phases. **(C-D)** Western blot analysis of cell cycle regulatory proteins in LN229 and U251 cells after 24 hours treatment with nebivolol. β-actin was used as a loading control. Data are presented as mean ± SD. Statistical significance was determined by comparison with the control group (*p < 0.05, **p < 0.01).

To further investigate the mechanism by which nebivolol inhibits GBM cell proliferation, we treated LN229 and U251 cells with different concentrations of nebivolol (5 and 10 µM) for 24 hours and analyzed the cell cycle distribution using flow cytometry. Compared to the control group, treatment with nebivolol significantly increased the percentage of cells in the G1 phase while reducing the number of cells in the G2 and S phases. Specifically, after treatment with 5 µM nebivolol, the proportion of LN229 and U251 cells in the G1 phase increased to 57.2% and 60.8%, respectively. Following treatment with 10 µM nebivolol, the G1 phase proportion further increased to 65.9% and 69.3% for LN229 and U251 cells, respectively (Fig 3A and 3B). These results indicate that nebivolol can arrest the cell cycle of GBM cells in the G0/G1 phase.

To validate these findings, we performed Western blot analysis. The expression levels of key cell cycle proteins, including CDK6, cyclin B1, and cyclin D1, were reduced in a dose-dependent manner in LN229 and U251 cells (Fig 3C and 3D). These results demonstrate that nebivolol effectively exerts its anti-proliferative effects on GBM cells by inducing cell cycle arrest at the G0/G1 phase.

### Nebivolol suppresses ATP synthesis and enhances mitochondrial membrane potential by inhibiting complex I activity in GBM cells

**Fig 4:**
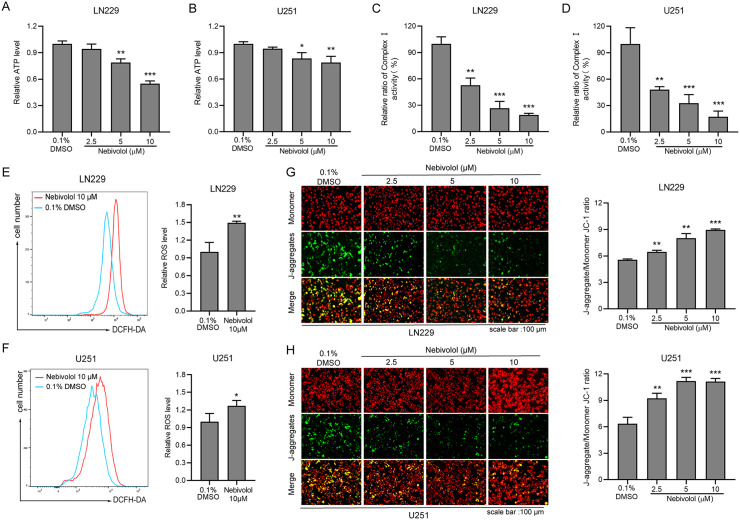
Nebivolol disrupts mitochondrial function by inhibiting complex I activity, elevating ROS production, and impairing ATP synthesis. **(A-B)** Quantification of intracellular ATP levels in LN229 (A) and U251 (B) cells treated with nebivolol for 24 hours, measured using a GloMax Luminometer. Data are expressed as a percentage relative to the vehicle control (0.1% DMSO). **(C-D)** Assessment of mitochondrial respiratory chain complex I activity in LN229 (C) and U251 (D) cells following 24 hours treatment with increasing concentrations of nebivolol. Complex I activity was normalized to the vehicle control group. **(E-F)** Flow cytometric analysis of intracellular ROS levels in LN229 (E) and U251 (F) cells treated with 10 μM nebivolol for 24 hours. Representative histograms depict the shift in fluorescence intensity, and quantitative data are expressed as fold change relative to the control. **(G-H)** Evaluation of mitochondrial membrane potential (ΔΨm) using JC-1 staining in LN229 (G) and U251 (H) cells treated with nebivolol for 24 hours. Fluorescence microscopy images show JC-1 monomers (green fluorescence) and aggregates (red fluorescence), scale bar = 100 μm. The ratio of red to green fluorescence intensity, quantified using a microplate reader. Data are presented as mean ± SD. Statistical significance was determined by comparison with the vehicle control group (*p < 0.05, **p < 0.01, ***p < 0.001).

Cellular growth relies on a continuous supply of ATP, and inhibition of ATP synthesis can effectively suppress tumor cell proliferation. To investigate whether nebivolol affects ATP synthesis in GBM cells, LN229 and U251 cells were treated with nebivolol for 24 hours, followed by quantification of intracellular ATP levels using the CellTiter-Glo 3D Cell Viability Assay. The results demonstrated that nebivolol treatment significantly reduced intracellular ATP levels in a dose-dependent manner (Fig 4A and 4B).

To further determine whether nebivolol acts as an inhibitor of mitochondrial complex I, we assessed complex I activity using the CheKine Mitochondrial Respiratory Chain Complex I Activity Assay, based on the principle that complex I catalyzes the dehydrogenation of NADH to NAD ⁺ . Notably, complex I activity in LN229 and U251 cells decreased significantly with increasing concentrations of nebivolol (Fig 4C and 4D).

Mitochondrial complex I is a major source of ROS in cells. Under physiological conditions, ROS are essential for cellular signaling and growth; however, excessive ROS production can cause cellular damage, increase the risk of DNA damage, and accelerate apoptosis. To elucidate the link between nebivolol-induced apoptosis and its anti-tumor activity, intracellular ROS levels were measured using the DCFH-DA probe and flow cytometry. Treatment with 10 µM nebivolol for 24 hours significantly increased ROS levels in LN229 and U251 cells (Fig 4E and 4F). Furthermore, JC-1 staining revealed that nebivolol treatment markedly elevated the mitochondrial membrane potential (ΔΨm) in GBM cells in a dose-dependent manner (Fig 4G and 4H). In summary, nebivolol inhibits mitochondrial complex I activity, reduces ATP synthesis, and increases mitochondrial membrane potential and ROS levels, thereby exerting its anti-tumor effects in GBM cells.

### Nebivolol inhibits tumor cell growth via the AKT/mTOR/4EBP1 signaling pathway in GBM cells

**Fig 5:**
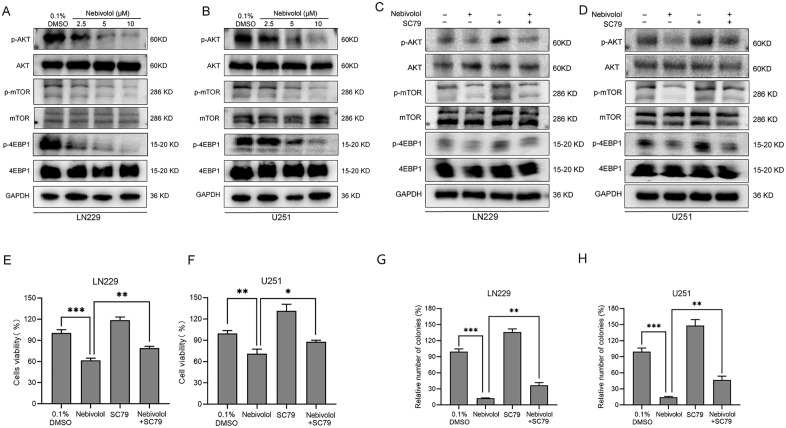
Nebivolol suppresses GBM cell proliferation through inhibition of the AKT/mTOR/4EBP1 signaling axis. **(A-B)** Western blot analysis of AKT/mTOR/4EBP1 pathway-related proteins in LN229 and U251 cells treated with increasing concentrations of nebivolol for 24 hours. **(C-D)** The effects of nebivolol (10 μM) and/or SC79 (10 μM) (an AKT activator) treatment for 24 hours on the levels of p-AKT, AKT, p-mTOR, mTOR, p-4EBP1 and 4EBP1 in LN229 and U251 cells were assessed by Western blot analysis. **(E-F)** Cell viability assessed by CCK-8 assay in LN229 and U251 cells treated with nebivolol (10 μM) alone or in combination with SC79 (10 μM) for 24 hours. Data are expressed as a percentage relative to the vehicle control (0.1% DMSO). **(G-H)** Colony formation assay in LN229 and U251 cells treated with nebivolol (10 μM) alone or in combination with SC79 (10 μM) for 24 hours. The number of colonies was normalized to the vehicle control group. Data are presented as mean ± SD of three independent experiments. Statistical significance was determined by comparison with the vehicle control group (*p < 0.05, **p < 0.01, ***p < 0.001).

The aberrant activation of the AKT/mTOR/4EBP1 signaling pathway is a critical contributor to GBM development and excessive proliferation. Previous studies have shown that blocking this pathway can inhibit GBM cell proliferation and induce apoptosis. In this study, we used Western blot analysis to examine the changes in key signaling molecules of the AKT pathway in LN229 and U251 cells treated with nebivolol. The results demonstrated that the phosphorylation levels of AKT, mTOR, and 4EBP1 decreased in a dose-dependent manner following nebivolol treatment in both GBM cell lines (Fig 5A and 5B), indicating that nebivolol significantly inhibits the activity of the AKT pathway in these cells.

To further confirm whether the AKT/mTOR/4EBP1 signaling pathway mediates the inhibitory effect of nebivolol on GBM cell growth, we treated GBM cells with SC79, an AKT pathway activator. Western blot analysis showed that SC79 partially reversed the inhibitory effects of nebivolol on the phosphorylation levels of AKT and its downstream proteins (Fig 5C and 5D). Using the CCK-8 assay, we found that co-treatment with nebivolol and SC79 partially reversed the inhibitory effects of nebivolol on the AKT/mTOR pathway in LN229 and U251 cells after 24 hours (Fig 5E and 5F). In colony formation assays, SC79 treatment also partially reversed the suppression of colony formation induced by nebivolol (Fig 5G and 5H). In conclusion, nebivolol inhibits GBM cell proliferation by partially blocking the AKT/mTOR/4EBP1 signaling pathway.

### Nebivolol significantly inhibits the growth of GBM xenografts *in vivo* and prolongs the survival of tumor-bearing mice

**Fig 6:**
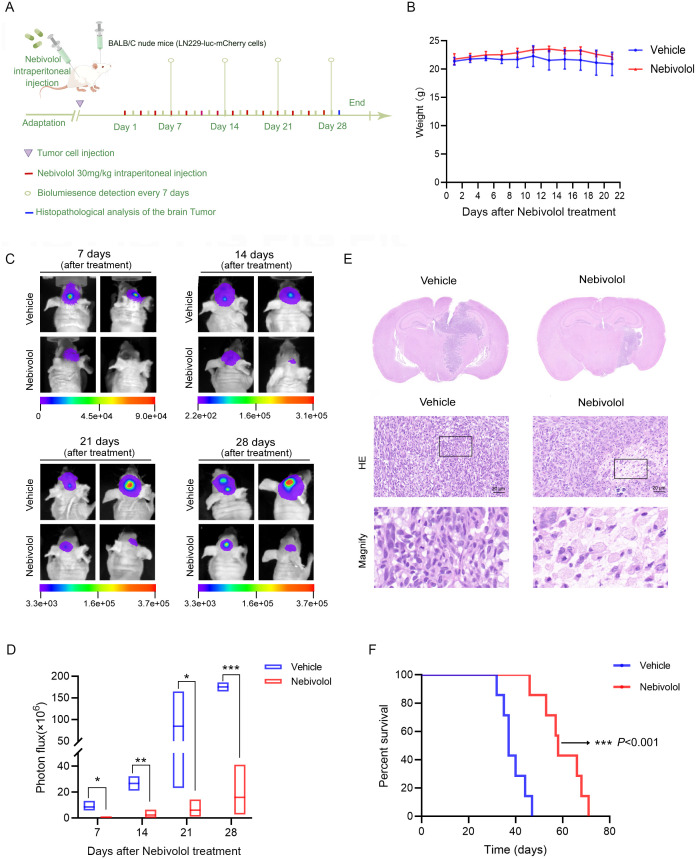
Nebivolol suppresses glioblastoma growth and prolongs survival in an orthotopic xenograft mouse model. **(A)** Schematic diagram of the experimental design for *in vivo* evaluation of nebivolol efficacy. **(B)** Body weight changes in mice monitored over the course of nebivolol treatment. **(C)** Representative *in vivo* bioluminescence images of tumor-bearing mice at indicated time points post-treatment. **(D)** Quantitative analysis of tumor burden based on bioluminescence photon flux (photons/sec/cm²/sr). **(E)** Representative images of histopathological analysis of whole brain sections stained with hematoxylin and eosin **(H&E)**, scale bar: 20 μm. **(F)** Kaplan-Meier survival curves showing significantly prolonged overall survival in nebivolol-treated mice compared to the vehicle control group (log-rank test, *p* < 0.001). Data are *p*resented as mean ± SD from three independent experiments. Statistical significance was determined by comparison with the vehicle control group (*p < 0.05, **p < 0.01, ***p < 0.001).

To validate the anti-tumor efficacy of nebivolol against GBM *in vivo*, we established an orthotopic xenograft model using LN229 cells expressing luc-mCherry. Seven days post-implantation, mice were randomly divided into groups, and treatment was initiated. Tumor growth was dynamically monitored using an *in vivo* imaging system on days 7, 14, 21, and 28 post-treatments, with fluorescence intensity recorded. On day 29, three mice from each group were euthanized, and brain tissues were collected for H&E staining to assess tumor volume. The remaining seven mice per group were used for survival analysis, as outlined in Fig 6A. Results demonstrated that intracranial tumor volume in the nebivolol-treated group was significantly smaller than that in the control group (Fig 6C and 6D), consistent with HE staining findings (Fig 6E). Survival analysis revealed that nebivolol treatment significantly extended the median survival of tumor-bearing mice by 21 days compared to the control group (Fig 6F), with statistical significance. Additionally, nebivolol treatment did not adversely affect the body weight of the mice (Fig 6B). In conclusion, nebivolol effectively suppresses intracranial GBM growth and significantly prolongs the survival of tumor-bearing mice.

## Discussion

Glioblastoma is the most common high-grade primary intracranial malignancy, characterized by a poor prognosis. Given the limited therapeutic options currently approved for GBM, there is an urgent need for new treatment strategies [5,27]. Although tumor cells exhibit enhanced glycolysis, mitochondrial OXPHOS remains essential for tumor growth [5–7,28]. Activation of β-adrenergic receptors in the cAMP/PKA signaling pathway promotes the phosphorylation of mitochondrial respiratory chain proteins and ATP synthase inhibitors, subsequently activating OXPHOS [7]. Nebivolol, an FDA-approved small-molecule compound, can penetrate the BBB and exert pharmacological effects intracranially [29,30]. Consistent with previous findings that β-blockers reduce glioma cell proliferation, migration, and enhance chemosensitivity [21], our study demonstrates that the selective β1-AR antagonist Nebivolol exerts anti‑GBM effects through blocking AKT/mTOR/4EBP1 signaling pathway. These results further support the translational potential of targeting β1-AR in glioblastoma therapy.

ROS have emerged as therapeutic targets in many types of tumors. Anti-tumor drugs, such as anthracyclines and topoisomerase inhibitors (e.g., doxorubicin, daunorubicin, and epirubicin), block DNA synthesis, inhibit topoisomerase II activity, and inactivate mitochondrial complexes I and II, thereby increasing mitochondrial ROS production to kill tumor cells [31,32]. Similarly, platinum-based drugs, including cisplatin, carboplatin, and oxaliplatin, induce tumor cell death by maintaining elevated ROS levels [33,34]. Notably, nebivolol specifically targets tumor cells without affecting isolated mitochondria or non-tumor cells, excluding mitotic toxicity [35]. In our study, nebivolol treatment significantly increased ROS levels, which in turn induced apoptosis in GBM cells. Animal experiments further confirmed that nebivolol markedly inhibited intracranial tumor growth and prolonged the median survival of tumor-bearing mice. Nebivolol has been reported to have a favorable safety profile in both clinical studies and preclinical animal models [30,36]. Nebivolol did not affect normal body weight or grooming behavior in mice, with only a modest reduction in heart rate at the highest dose and no significant effect on mean arterial pressure. Consistent with these findings, we observed no significant alterations in body weight, activity, or overall well-being in our treated mice throughout the experiment. These results suggest that nebivolol holds promise as a potential therapeutic agent for GBM, providing a foundation for future clinical trials.

Tumor cell apoptosis occurs through intrinsic and extrinsic pathways. The intrinsic pathway, initiated by mitochondrial signals, involves stimuli such as DNA damage and growth factor deprivation. These stimuli activate Bax/Bak oligomer complexes, which insert into the mitochondrial outer membrane, alter mitochondrial permeability, and promote cytochrome c release into the cytoplasm. Cytochrome c then binds with Apaf-1 to form the apoptosome, activating caspase-9 and subsequently caspase-3 and caspase-7, triggering a caspase cascade that culminates in apoptosis [37]. The PI3K/AKT signaling pathway plays a critical role in cellular processes and has been found to be abnormally activated in hepatocellular carcinoma, lung cancer, ovarian cancer, and GBM [38–41]. A critical mechanism by which the AKT pathway mediates apoptosis inhibition is through the regulation of Bax and Bcl-xl [42]. The dynamic balance between the pro-apoptotic protein Bax and the anti-apoptotic protein Bcl-xl governs the progression of mitochondria-mediated apoptosis [43]. AKT phosphorylates Bax at Ser184, preventing its mitochondrial membrane insertion and inhibiting its pro-apoptotic activity [42]. Additionally, PI3K/AKT activation upregulates Bcl-xl expression, suppressing GBM cell apoptosis [44]. Consistent with those results, in our study, we found that nebivolol treatment blocked AKT/mTOR pathway activation, leading to increased BAX and decreased BCL-2 expression, thereby promoting apoptosis. Conversely, activating the AKT/mTOR signaling pathway reversed the inhibitory effect of nebivolol on GBM proliferation.

In this study, we observed that nebivolol increased mitochondrial membrane potential, consistent with findings from Cristina Nuevo-Tapioles et al., who demonstrated that oligomycin (an ATP synthase inhibitor) exerted similar effects in colorectal and breast cancer cells [7]. Studies have shown that the loss of ATPIF1 maintains ΔΨm to inhibit antimycin-induced cell death [45]. Under conditions of OXPHOS deficiency, cells initiate adaptive mechanisms to maintain ΔΨm [46], including the downregulation of ATP synthase inhibitors and OXPHOS subunits, Snf1/AMPK-mediated glycolytic upregulation, inhibition of ribosome biogenesis, and upregulation of cytoplasmic chaperones. These adaptations inhibit mitochondrial ATP hydrolysis, remodel the mitochondrial proteome, and optimize ATP supply to sustain ΔΨm, ISC biosynthesis, and cell proliferation [47–49]. Based on our experimental results, we hypothesize that the nebivolol-induced increase in ΔΨm may result from adaptive responses to mitochondrial respiration inhibition rather than direct mitochondrial proton retention.

**Fig 7:**
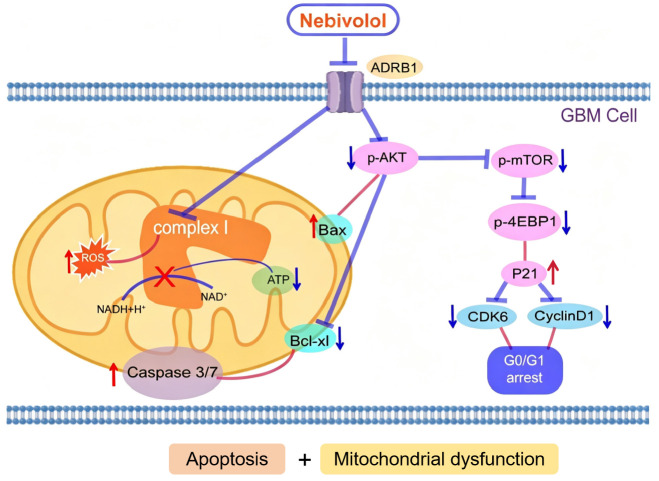
Working model. Nebivolol inhibits β1-adrenergic signaling, leading to suppression of mitochondrial complex I activity, disruption of oxidative phosphorylation, and subsequent metabolic reprogramming characterized by elevated ROS production and reduced ATP synthesis, ultimately triggering intrinsic apoptosis. Concurrently, Nebivolol inactivates the AKT/mTOR signaling axis, inducing cell cycle arrest at the G0/G1 phase and inhibiting proliferation, thereby promoting tumor cell death.

Our data further demonstrate that the β1-adrenergic blocker nebivolol suppresses GBM growth by affecting mitochondrial metabolism and blocking the AKT signaling pathway. Notably, nebivolol exerts its effects by inhibiting mitochondrial complex I activity, leading to mitochondrial dysfunction, ROS accumulation, and partial blockade of the AKT/mTOR/4EBP1 signaling pathway, ultimately exerting anti-tumor effects in GBM (Fig 7). In conclusion, our findings support targeting mitochondrial metabolism as a potential and promising strategy for GBM treatment and provide a theoretical foundation for future clinical trials of nebivolol.

Some limitations should be considered in our study. Given that commercially available glioma cell lines do not fully capture the heterogeneity of patient tumors, validation using patient-derived primary glioma cells is therefore essential to strengthen the clinical relevance of our findings. Although nebivolol is a selective ADRB1 antagonist, the potential involvement of ADRB2 or non-adrenergic receptor pathways in its anti-glioma activity warrants further investigation.

## Materials and Methods

### Ethics statement

The experimental protocol for animal studies was reviewed and approved by ethics committee of Xuzhou Medical University

### Culture of cell lines

Human GBM cell lines (LN229, U251, T98G, and U87) and normal human astrocyte cell line (HA1800) used in this study were obtained from the Shanghai Cell Bank, Chinese Academy of Sciences. These cell lines were cultured and maintained in Dulbecco’s Modified Eagle Medium (DMEM) (KeyGEN BioTECH, Nanjing, China) supplemented with 10% fetal bovine serum (FBS) and grown in a 37 °C moist incubator containing 5% CO_2_.

### Reagents and antibodies

Nebivolol and AKT activator SC79 were purchased from MedChemExpress (Shanghai, China). Nebivolol and SC79 were dissolved in DMSO to generate stock solution (10 mM), which was diluted to different concentrations in DMEM medium before use.

Bax (#2772), Bcl-xl (#2762), p21 (#2947), p27 (#3686), cyclin D1 (#2922), cyclin B1 (#12231), CDK6 (#3136), AKT (#9272), p-AKT (#4058), p-mTOR (#5536), mTOR (#2983), p-4EBP1 (#2855), 4EBP1 (#9644), GAPDH (#5174) and β-actin (#8457) primary antibodies were purchased from Cell Signaling Technology (CST, MA, USA).

### CCK-8 assay

Cell counting kit-8 (CCK-8, Vicmed, Jiangsu, China) was used for evaluating cell viability. Briefly, U87, T98G, LN229, U251 and HA1800 cells were seeded in 96-well plates at a density of 4 × 10^3^ cells per well, with 3 replicate wells set for each group. After cell adhesion, different concentrations of nebivolol (0–20 µM) were added to the experimental group, and 0.1% DMSO was added to the control group. The treatment lasted for 72 hours. 100 µL of medium containing 10% CCK-8 was added to each well. After incubation in the dark for 30 minutes, the absorbance was detected at a wavelength of 450 nm using a microplate reader.

### Colony formation assay

LN229 and U251 cells were plated in 6-well plates at a density of 1 × 10^3^ cells per well. After cell adhesion, nebivolol was added to the experimental group and 0.1% DMSO was added to the control group. After 24 hours of treatment, the medium was replaced with fresh medium without nebivolol and the cells were further cultured for 10 days. The cells were washed with PBS and fixed with 4% paraformaldehyde for 30 minutes, stained with crystal violet working solution for 30 minutes, the staining solution was washed away, clones were observed, photographed, and counted for statistical analysis.

### EdU incorporation assay

Cell proliferation was detected by using EdU cell proliferation detection kit (Abbkine, Hubei, China). LN229 and U251 cells were plated in 96-well plates at a density of 6 × 10^3^ cells per well. After cell adhesion, the experimental group was treated with Nebivolol for 24 hours. The cells were incubated with 10 μM EdU for 2 hours and then fixed with 4% paraformaldehyde for 30 minutes. After PBS washing, the cells were treated with 0.5% Triton X-100 for 10 minutes. Finally, the cells were incubated with 100 μL Click-iT for 30 minutes and stained with DAPI for 15 minutes. After three washes with PBS, the cells were photographed under a fluorescence inverted microscope.

### Cell cycle and apoptosis detection

LN229 or U251 cells were plated in 6 cm culture dishes. After cell adhesion, Nebivolol was added to the experimental group. After 24 hours of treatment, the cells were collected. The cells were fixed with 70% ice-cold ethanol overnight, washed twice with PBS, and stained with a solution containing PI/RNase for 15 minutes.

For cell apoptosis, the cells were washed twice with cold PBS and stained with the Annexin V-FITC/PI apoptosis detection kit. Flow cytometry was used for detection and flow cytometry software was used for analysis of the cell cycle distribution and apoptosis.

### Western blot assay

The experimental group was treated with nebivolol on LN229 and U251 cells, and total proteins were collected after 24 hours. The protein concentration was determined by the Bradford method and the immunoblotting assay was performed. The expression levels of AKT, p-AKT, mTOR, p-mTOR, 4EBP1, p-4EBP1, CDK6, Bax, Bcl-xl, cyclinD1, and cyclinB1 were analyzed, with GAPDH and β-actin as internal reference controls.

### ATP level detection

LN229 and U251 cells were seeded in 96-well white plates. After cell adhesion, the cells were treated with 0.1% DMSO and Nebivolol for 24 hours. 10 μL CellTiter-Glo buffer was added to the control group and the treatment group and mixed evenly. After incubation at room temperature in the dark for 10 minutes, the fluorescence value of each sample was detected using the GloMax Luminometer.

### ROS level detection

LN229 and U251 cells were plated in 6 cm culture dishes at a density of 100,000 cells per dish. After treatment with 0.1% dimethyl sulfoxide (DMSO) or 10 µM nebivolol for 24 hours, the DCFH-DA probe was added and incubated for 30 minutes. The cells were washed twice with phosphate-buffered saline (PBS) to remove any probe that did not enter the cells. A positive control, Rosup, was included, and the cells were incubated at 37°C for 30 minutes. The cells were then trypsinized to a single-cell suspension, and the digestion was terminated using serum-free medium. The cells were transferred to flow tubes and analyzed by flow cytometry. The rate of ROS-positive cells was determined using FlowJo software (version 10).

### Mitochondrial respiratory chain complex I activity assay

LN229 and U251 cells were treated with 0.1% DMSO or varying concentrations of nebivolol for 24 hours. The activity of mitochondrial complex I was measured using a mitochondrial respiratory chain complex I activity detection kit (Abbkine, Hubei, China) according to the manufacturer’s instructions. The initial absorbance (A1) at 0 minutes and the absorbance (A2) at 2 minutes were measured at 340 nm using spectrophotometry. The change in absorbance (ΔA) was calculated as ΔA = A1 - A2. Complex I activity (U/10⁴ cells) was determined using the formula: complex I activity = 1.46 × ΔA. One unit (U) of enzyme activity was defined as the consumption of 1 nmol NADH per 10,000 cells per minute. The values were recorded and statistically analyzed.

### Mitochondrial membrane potential assay (JC-1)

LN229 and U251 cells were treated with 0–10 µM nebivolol for 24 hours, and changes in mitochondrial membrane potential were assessed using the JC-1 Mitochondrial Membrane Potential Assay Kit. The culture medium was aspirated, and the cells were washed three times with PBS. The JC-1 dye was thawed at 4°C and diluted in culture medium to a final concentration of 2 µM. A 100 µL aliquot was added to each well, and the cells were incubated at 37 °C for 15 minutes in the dark. The cells were washed twice with 1 × buffer, and fresh culture medium was added. Fluorescence images were captured using a fluorescence microscope.

For quantitative analysis, a microplate reader was used. The excitation wavelength was set to 528 nm, and the emission wavelength was set to 560 nm to measure the fluorescence intensity of JC-1 aggregates (red fluorescence). The excitation wavelength was adjusted to 485 nm, and the emission wavelength was set to 530 nm to measure the fluorescence intensity of JC-1 monomers (green fluorescence). The fluorescence values were statistically analyzed. FCCP, a mitochondrial uncoupler, was used as a positive control.

### Animal experiments

All animal protocols in this study were approved by the ethics committee of Xuzhou Medical University. Male BALB/c athymic nude mice (6 weeks old, 20–23 g) were obtained from Weitong Lihua Experimental Animal Technology Co., Ltd. (Beijing, China). LN229 cells expressing luc-mCherry were orthotopically injected into the right intracranial region of 20 mice, with 1.5 × 10⁶ cells injected per mouse. Seven days after tumor cell implantation, the mice were randomly divided into two groups: a vehicle control group (0.9% NaCl) and a nebivolol treatment group (30 mg/kg, dissolved in 0.9% NaCl), with 10 mice per group. The drugs were administered intraperitoneally every two days. Three mice from each group were randomly selected, and tumor growth was dynamically monitored using bioluminescence imaging with a small animal imaging system. Images were captured once per week. Four weeks later, the mice were perfused, and brain tissues were collected for H&E staining to assess tumor size. The remaining seven mice in each group were used for survival analysis. Mice exhibiting neurological symptoms, such as rotational behavior, reduced activity, or domed head, were euthanized, and survival times were recorded.

### Statistical analysis

All experiments were independently repeated at least three times, and the presented figures are representative of one of the repeated experiments. Data were analyzed using GraphPad Prism 7.0 and are presented as mean ± standard deviation (SD). Comparisons between two groups were performed using an independent samples t-test, while comparisons among more than two groups were analyzed using one-way ANOVA. Survival outcomes were evaluated using the Kaplan-Meier method, and differences in survival between groups were assessed using the log-rank test. A significance level of α = 0.05 was used, and results with *P < 0.05 were considered statistically significant.
